# Genetic Characterization of the Gypsy Moth from China (Lepidoptera, Lymantriidae) Using Inter Simple Sequence Repeats Markers

**DOI:** 10.1371/journal.pone.0073017

**Published:** 2013-08-07

**Authors:** Fang Chen, Juan Shi, You-qing Luo, Shuang-yan Sun, Min Pu

**Affiliations:** 1 Forestry College, Beijing Forestry University, Beijing, China; 2 Standards and Regulations Research Center of AQSIQ, Beijing, China; University of Guelph, Canada

## Abstract

This study provides the first genetic characterization of the gypsy moth from China ( *Lymantria* *dispar* ), one of the most recognized pests of forests and ornamental trees in the world. We assessed genetic diversity and structure in eight geographic populations of gypsy moths from China using five polymorphic Inter simple sequence repeat markers, which produced reproducible banding patterns. We observed 102 polymorphic loci across the 176 individuals sampled. Overall genetic diversity (Nei’s, H) was 0.2357, while the mean genetic diversity within geographic populations was 0.1845 ± 0.0150. The observed genetic distance among the eight populations ranged from 0.0432 to 0.1034. Clustering analysis (using an unweighted pair-group method with arithmetic mean and multidimensional scaling), revealed strong concordance between the strength of genetic relationships among populations and their geographic proximity. Analysis of molecular variance demonstrated that 25.43% of the total variability (*F*_*ST*_ = 0.2543, *P < 0.001*) was attributable to variation among geographic populations. The results of our analyses investigating the degree of polymorphism, genetic diversity (Nei’s and Shannon) and genetic structure, suggest that individuals from Hebei may be better able to adapt to different environments and to disperse to new habitats. This study provides crucial genetic information needed to assess the distribution and population dynamics of this important pest species of global concern.

## Introduction

The gypsy moth, *Lymantria* *dispar* Linnaeus (Lepidoptera: Lymantriidae), is a destructive defoliator with a broad host range, and one of the most recognized pests of forests and ornamental trees in the world. Indeed, it is the only forest insect to be included in the American Quarantine regulations [1]. The gypsy moth has been divided into two subspecies, the European gypsy moth (EGM), *Lymantria* *dispar* *dispar* and the Asian gypsy moth (AGM), *Lymantria* *dispar* *asiatica*, based on preferred host plant usage and the females’ flight abilities. The EGM was originally found in Europe, but was accidentally introduced into North America from France in 1869 [2], where it has remained ever since. The AGM is primarily found in Asia, but also exists in some European areas. The AGM is considered to pose a more significant threat globally, owing to its preference for a broader range of host species [3] and to the higher flight capability of females [4]. According to historical materials, in China, the core distribution of the AGM lies between 20^o^ and 58^o^ north, covering an area of more than 20 provinces or autonomous regions [5]. The AGM’s ability to survive on more than 500 host plants, including numerous conifers, as well as broadleaf trees and fruit trees, results in severe damage to domestic tourism and the forestry economy [6].

Genetic variability is a crucial indicator of the capacity of a species or population capacity to adapt to the environment [7]. Species or populations with higher genetic variability are better equipped to adapt to novel environments, enabling an greater capacity for range expansion [8]. Information about genetic variation can also provide scientific evidence for exploring a species’ origin, its geographic spread and implementing targeted quarantine measures.

Previous research using microsatellite DNA markers to assess genetic variability in four gypsy moth populations from Japan, eastern Russia, China, and North America revealed that genetic variation in the North American population was lower than that in the Asian populations [9]. Amplified fragment length polymorphism (AFLP) analysis on genetic variability of populations from Europe, Asia and North America, on the other hand, indicated that EGM populations have experienced historic population bottlenecks as a consequence of past environmental changes [10]. Genetic analyses based on three mitochondrial gene regions, meanwhile, revealed that there was extensive genetic variation among Japanese populations of gypsy moths, with three haplogroups identified (a: Okinawa, b: Hokkaido, and c: Honshu, Kyushu and mainland Asia), while samples from Europe, Tunisia and North America grouped to form a fourth distinct haplogroup [11]. More recently, a global bar code library was constructed for tussock moth based on the cytochrome oxidase I sequence, which indicated that there existed clear divergence between the *L* . *d* *. dispar* subspecies and the two Asian subspecies *asiatica* and *japonica* [12]. In all the studies listed above, the gypsy moth from China was treated as a single strain. However, no systematic research has been conducted on the Chinese AGM and, hence, we have no knowledge of the genetic structure and variability within Chinese populations.

In this study, we assessed the genetic diversity of the gypsy moth within China using Inter Simple Sequence Repeat (ISSR) markers. The ISSR technique entails the amplification of DNA fragments located between two closely and inversely oriented microsatellites [13]. Primers consist of a short microsatellite sequence (16–18 bases) with 1–3 non-repeated anchoring bases [14]. ISSR amplification does not require prior knowledge of a species’ genome and produces multilocus and highly polymorphic patterns [15,16]. ISSR is a simple, inexpensive, nonradioactive, reproducible and powerful tool for assessing genetic diversity [17–20].

## Materials and Methods

### Sample collection and animal rearing

**Figure 1:**
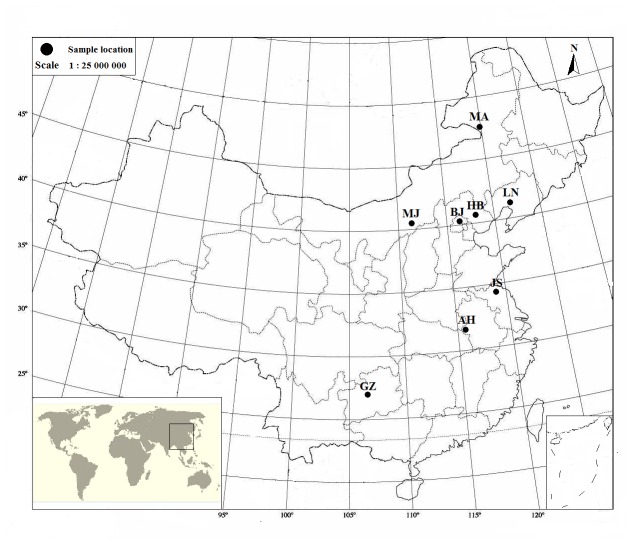
Locations of 8 *L* *. dispar* sampling sites in China.

A total of 176 specimens of *L* *. dispar* sourced from eight locations (Figure 1) in China were analyzed in this study (Table 1). Permission for sample collection was authorized by Forest Protection Station of China’s National Forestry Bureau and the process of each location was accompanied by staff of the local forestry bureau. With the exception of the nine adult moths (6 male, 3 female) from Jining, Inner Mongolia, which were captured by hand in the field, all the others were reared to mature larvae from egg masses on an artificial diet supplied by the Chinese Academy of Forestry Sciences in illuminated incubators, which maintained ambient conditions at 25±0.5^o^C, 40-50% RH, and a photoperiod of L:D=16:8 h [4]. Prior to use, moths were starved for 24 hours, and then frozen at -80^°^ C.

**Table 1:** Details of the gypsy moth samples collected and analyzed in this study.

Accession code	Collection site	Sample size	Latitude	Longitude	Collection date
AH	Liu’an, Anhui	30	31°30′34″	115°53′39″	Aug, 2011
BJ	Yanzikou, Beijng	30	40°19′21″	116°09′11″	Aug, 2011
GZ	Xifeng, Guizhou	20	27°11′40″	106°49′53″	Nov, 2011
HB	Longhua, Hebei	24	41°37′88″	118°14′49″	Mar, 2012
JS	Lianyungang, Jiangsu	29	34°44′16	119°22′45″	Jul, 2011
LN	Sandeli, Liaoning	24	41°30′33″	122°22′27″	Sep, 2011
MA	Arxan, Inner Mongolia	10	47°10′26″	119°56′44″	Jun, 2011
MJ	Jining, Inner Mongolia	9	40°36′47″	112°07′30″	Aug, 2011

### DNA extraction

Approximately 30 to 50 mg body tissue was detached from each individual for DNA extractionfollowing the manufacturer’s instructions of the Insect gDNA Miniprep Kit (BIOMIGA, Beijing, China). Compared to traditional insect DNA extraction methods, such as SDS [21] and CTAB [22], the kit protocol removed salts, proteins and other contaminants more thoroughly, thus higher and purer genomic DNA (gDNA) was produced. Then, concentration and purity of extracted gDNA was assayed by a Spectrophotometer ND1000 V 3.5.2 (Gene Company Limited, Hong Kong, China), A260/A280 values between 1.8 and 2.0 were deemed of sufficient quality. 50ng/µL gDNA was preserved at -20°C until future use.

### Screening of ISSR PCR primers

Initially 105 ISSR primers were used to amplify three random DNA samples, of which one hundred were designed by the biotechnology laboratory of the University of British Columbia [23] and the other five which were employed in *Aristaeomorpha foliacea* ISSR work [13]. Thirty-seven of these primers successfully amplified *L* *. dispar* DNA; however, twenty-seven just produced single patterns. Ultimately, five ISSR primers capable of producing reproducible and unambiguous multiple (listed in Table 2) were used in all analyses reported in this study.

**Table 2:** List of ISSR primers used for genetic analyses of *L* *. dispar* .

Primer	Sequence (5’–3’)	Tm^a^ (ºC)	Ta^b^ (ºC)	Size range (bp)	Number of multiple bands
UBC 818	CACACACACACACACAG	54.59	53.2	280-2000	22
UBC 847	CACACACACACACACAR ^c^C	55.02-57.30	47.1	300-2000	23
IT 1	CACACACACACACACAGT	55.02	46.0	280-2000	22
IT 2	CACACACACACACACAAC	55.02	54.9	400-1800	16
IT 3	CACACACACACACACAAG	55.02	49.4	400-2000	19

^a^ Melting temperature

^b^ Annealing temperature

^c^ R: A or G

### Inter Simple Sequence Repeat

All PCR amplifications were carried out in a 20 µL volume, containing 10 ng of template DNA, 2 µL 10× PCR buffer (Takara Bio, Beijing, China), 1 µM of primer (SBS Genetech Co., Ltd, Beijing, China), 240 µM of each dNTP (Takara Bio, Beijing, China), 1.5 mM of MgCl_2_ (Takara Bio, Beijing, China), 0.75 U Taq DNA Polymerase (Takara Bio, Beijing, China) and ddH_2_O to the final volume. The amplification reaction was performed in a DNA Engine Thermal Cycler PTC-200 (BIO-RAD, Shanghai, China). The conditions were 94^o^C for 5 min, 35 cycles of 94^o^C for 45 s, annealing at their respective temperature for 45 s and 72^o^C for 90 s, and final extension at 72^o^C for 10 min. For each primer, replicates and negative controls were included to ensure the consistency of results.

To analyze the PCR product, 5 µL of product was added to 1 µL of 6× loading buffer (Takara Bio, Beijing, China), and this mixture was loaded in a 2% agarose gel (Ffgene Co., LTD. Spain) for electrophoresis with 0.5× TAE (Tris/Acetic/EDTA) electrophoresis buffer. After running at 100 V for 1 h, the gel was stained in ethidium bromide (EB) buffer for 3 min, rinsed with distilled water and photographed in a gel documentation machine (Gene Company Limited, Hong Kong, China). A DL2000 DNA marker (Takara Bio, Beijing, China) was run as a standard with each primer.

### Statistical Analyses

The presence or absence of bands was scored as a 1 or a 0, thusour data was consisted by a presence/absence (1/0) matrix of 102 multiple bands from 176 individuals.

The program PopGene32 [24] was used to analyze genetic diversity, genetic identity (the proportion of genes that are identical in two populations) and genetic distance under assumptions of the Hardy-Weinberg equilibrium. To compare the genetic variability of each population, the genetic diversity of the different geographic populations was assessed by estimating the percentage of polymorphic loci (% P), Nei’s gene diversity [25] and Shannon’s Information index [26]. To reveal the genetic relationship among the eight populations, genetic identity and distance of eight *L* *. dispar* populations was obtained by Nei’s original measurements [27]. And a dendrogram of the eight different geographic populations was constructed based on Nei’s (1972) genetic distances using the UPGMA (unweighted pair-group method with arithmetic mean) method.

To examine the partitioning of genetic variance within and among samples from different geographical regions, Analysis of molecular variance (AMOVA) [28] was carried out in the program ARLEQUIN 3.5 [29] using 1/0 matrix. Genetic differentiation coefficients between populations were calculated as *F*_*ST*_, with 95% confidence intervals (CI) obtained by bootstrapping 1000 replicates over all loci.

## Results

Overall, 102 different bands, varying from 280 to 2,000 base pairs, were obtained by the five primers screened for 176 individuals across the eight geographic populations. All primers screened in this work were polymorphic, and the number of polymorphic bands per primer ranged from 16, for IT2, to 23, for UBC847. The primer which amplified the highest mean number of bands per population was IT1 (16.87 polymorphic bands); whereas that producing the lowest mean value was IT3 (9.25 polymorphic bands). Hebei was the geographic population with the highest number of bands (84 polymorphic bands); whilst Arxan, Inner Mongolia was the geographic population with the lowest mean number of bands (42 polymorphic bands; Table 3).

**Table 3:** Performance of each ISSR primer tested on *L* *. dispar* , with the number of specimens amplified (N_A_), the number that amplified for all primers (N_P_), and the number of polymorphic bands obtained for all (N_B_) and each primer (UBC818, UBC847, IT1, IT2 and IT3).

Pop ID	Number of specimens			Number of bands					
	N_A_	N_P_		N_B_	UBC818	UBC847	IT1	IT2	IT3
AH	30	30		74	12	18	17	13	14
BJ	30	30		58	10	17	17	8	6
GZ	20	19		67	10	14	17	10	16
HB	24	24		84	18	21	21	11	13
MJ	9	8		61	16	15	19	9	2
JS	29	27		57	12	16	13	9	7
LN	24	22		78	18	18	19	12	11
MA	10	7		42	7	12	12	6	5
Total	176	167		102					

**Figure 2:**
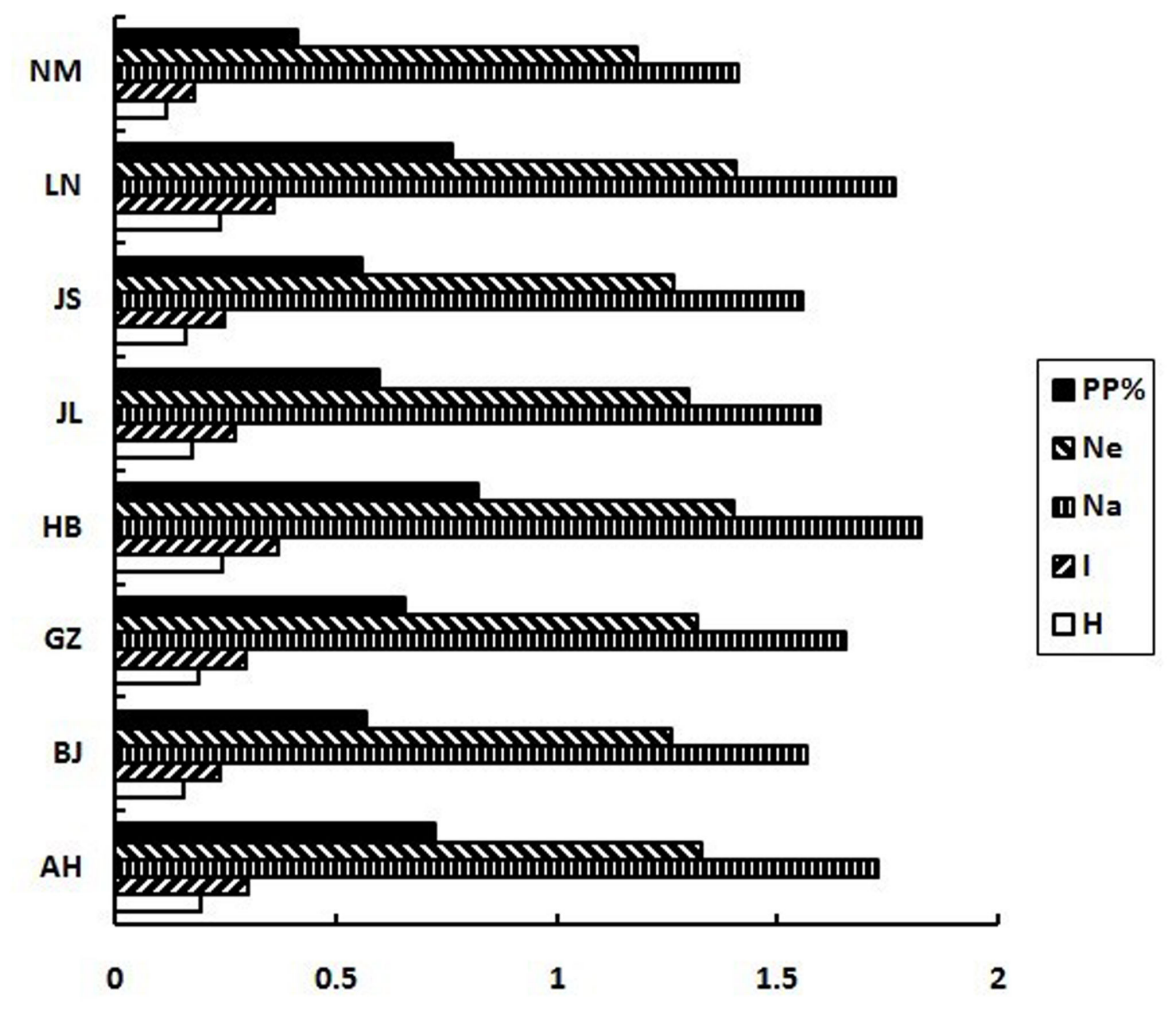
Comparison of genetic diversity of *L* *. dispar* in China. PP% is the percentage of polymorphic loci, Ne is the mean effective number of alleles, Na is the mean observed number of alleles, I is the mean Shannon’s information index and H is the mean Nei’s gene diversity.

The Nei’s gene diversity (H = 0.2357) and Shannon’s information index (I = 0.3735) showed relatively high overall genetic diversity for gypsy moth from China. Among the different geographic populations, Hebei showed the highest genetic diversity (H = 0.2410, I = 0.3707), while Arxan, Inner Mongolia showed the lowest (H = 0.1149, I = 0.1800) (Figure 2).

The values of genetic distance among the eight populations of *L* *. dispar* ranged from 0.0432 to 0.1034, suggesting a large genetic base (Table 4). The genetic distance between AH and JS (0.0432) revealed that these were the two most closely related geographic populations (Table 4). In contrast, the two most genetically distant geographic populations were GZ and MA (genetic distance value = 0.1034; Table 4).

**Table 4:** Values of genetic identity (above diagonal) and genetic distance (below diagonal) for eight *L* *. dispar* populations from China obtained by Nei’s (1972) original measures.

Pop ID	AH	BJ	GZ	HB	MJ	JS	LN	MA
AH	****	0.9373	0.9441	0.9363	0.9432	0.9577	0.9116	0.9345
BJ	0.0647	****	0.9364	0.9346	0.9328	0.9403	0.9323	0.9367
GZ	0.0575	0.0657	****	0.9402	0.9132	0.9400	0.9239	0.9018
HB	0.0659	0.0676	0.0617	****	0.9288	0.9313	0.9563	0.9213
MJ	0.0585	0.0695	0.0908	0.0739	****	0.9283	0.9128	0.9503
JS	0.0432	0.0616	0.0618	0.0712	0.0744	****	0.9168	0.9243
LN	0.0926	0.0701	0.0791	0.0447	0.0912	0.0868	****	0.9118
MA	0.0678	0.0654	0.1034	0.0819	0.0510	0.0787	0.0924	****

**Figure 3:**
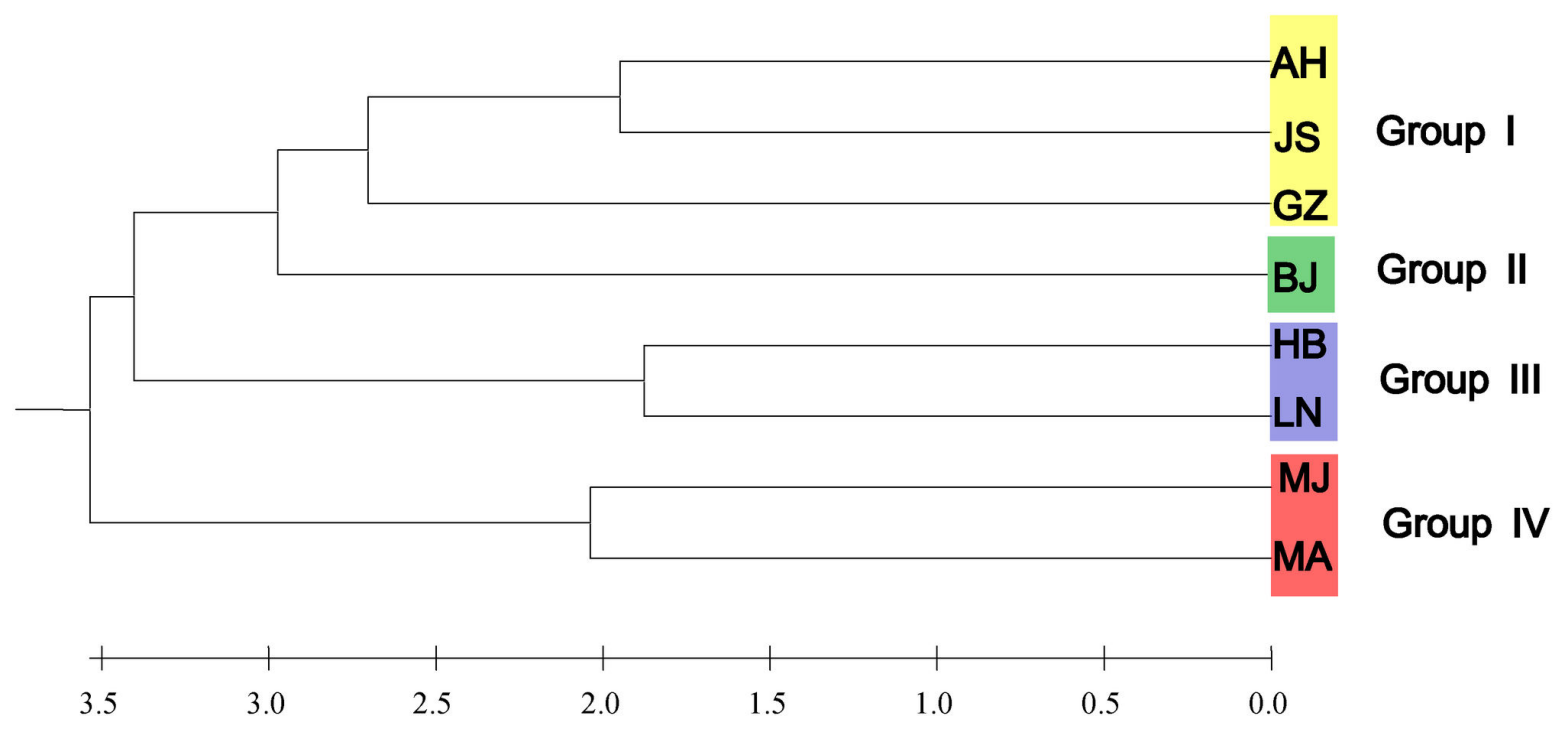
Dendrogram showing the genetic relationships among eight populations of gypsy moth from China with UPGMA method.

The UPGMA clustering analysis divided the eight populations of *L* *. dispar* into four groups at the threshold values of 2.75 (Figure 3). Group 1 contained the three southern Chinese populations (AH, GZ and JS), while Group 2 consisted solely of the Beijing (BJ) population. The four remaining northern Chinese populations were split between Groups 3 and 4. Group 3 consisted of the two more closely related populations with higher genetic diversity (HB & LN, genetic distance = 0.0447; genetic diversity [H] = 0.2410 and 0.2384 respectively). On the other hand, both the two populations from Inner Mongolia comprising Group 4 (MJ, MA) were more genetically dissimilar (genetic distance = 0.0510) and displayed lower genetic diversity (0.1769 and 0.1149 respectively).

AMOVA conducted on ISSR markers confirmed the presence of significant genetic differentiation among the eight geographic populations (with genetic variability among geographic populations accounting for 25.43% of the total variation: *F_ST_* = 0.2543, *P < 0.001*; Table 5). Gene flow (*Nm*) was estimated as 1.466 via the formula: *Nm* = (1- *F*_*ST*_)/2*F*_ST_ [30]. This value indicates moderate genetic connectivity among the populations and suggests that genetic drift does not contribute to population differentiation.

**Table 5:** Analysis of molecular variance (AMOVA) of *L* *. dispar* populations from China.

Source of variation	df	Sum of squares	Variance components	Percentage variation
Among populations	7	608.525	3.54466	25.43070
Within populations	168	1746.168	10.39386	74.56930
Total	175	2354.693	13.94423	-

## Discussion

The AGM has received close attention as a potential pest species over the past few decades because, unlike EGM, their eggs could complete diapause under a lower chill requirement [31], their larvae are capable of utilizing a broader range of host species [32] and their adults show higher flight ability [4]. According to historical reports in China, the AGM is primarily distributed in the northeast and northwest of the country. Although there existed report about gypsy moth’s occurrence in southern China in certain period, it turned out to be its sibling species such as *Lymantria* *xylina* Swinhoe and *Lymantria* *monacha* Linnaeus when monitoring with attractant the next years (personal communication with ZC. Xu, associate professor of Beijing Forestry University). To determine the genetic composition and structure of the gypsy moth from China, we conducted a large-scale analysis of genetic variability across eight different geographic populations, utilizing novel ISSR molecular markers [33]. This investigation is the first assessment of the efficiency of ISSR markers for estimating genetic diversity of *L* *. dispar* in China.

The efficiency of molecular markers is evaluated by the degree of polymorphism detected among the populations under investigation. All five primers, UBC818, U847, IT1, IT2 and IT3 used for analyses in this study were polymorphic. Unlike another previous genetic study of *L* *. dispar* with AFLP which demands of preliminary pedigree analysis, independent inheritance and alletic assignment of AFLP markers [10], the ISSR marker does not need prior knowledge of a species’ genome. In addition, the 102 polymorphic loci revealed by the five ISSR primers used in this study are much easier to score than the complex banding pattern derived from AFLP.

Polymorphism of a population depends on the existence of genetic variants, represented by the number of alleles per locus and by their frequency distribution in the population [34]. The results of our analysis of polymorphic loci within *L* *. dispar* populations from different localities, revealed that the Hebei population (HB) had the highest percentage of polymorphic loci (82.35%), suggesting this population could be the longest-existence of the eight populations or could be the largest population. Individuals from Arxan, Inner Mongolia (MA) showed the least genetic diversity, probably because of the frequent eradication measures undertaken by the local government every year.

Our analyses revealed high genetic diversity in the gypsy moth from China (as measured by both the Nei’s and Shannon’s index), which may be explained by the great dispersal capabilities at adult stage of AGM. Hebei (HB) had the highest genetic diversity of all the geographic populations (*H* = 0.2410, *I* = 0.3707), agreeing with the data for percentage of polymorphic loci. This suggests that individuals from Hebei possessing greater information content may be better able to adapt to different environments and, therefore, to disperse to new habitats.

Cluster analyses utilizing UPGMA revealed stronger genetic relationships among geographically proximate locations, suggesting that more closely related populations share a common origin, or evolutionary history. Our results also revealed high overall genetic differentiation among populations (AMOVA: *F*_*ST*_ = 0.2543, *P < 0.001*) lending further support to the finding of strong genetic structuring among the Chinese AGM. What is noteworthy is that there exists large degree of genetic separation among the five northern Chinese populations, which might be driven by the accumulation of genetic differences via adaptation of these populations to their differing environments.

In conclusion, this study has revealed that ISSR markers are a highly informative and efficient tool for estimating the genetic variation and structure of the AGM. The results of our analyses investigating the degree of polymorphism, genetic diversity (Nei’s and Shannon) and genetic relationships among different populations all indicate that individuals from Hebei pose the greatest threat as potential pests, and should be closely monitored by the phytosanitary department. The genetic information on the Chinese AGM obtained in this study will assist in elucidating crucial information on the distribution and population dynamics of this important pest species of global concern.
