# Correction: Genetic Characterization of the Gypsy Moth from China (Lepidoptera, Lymantriidae) Using Inter Simple Sequence Repeats Markers

**DOI:** 10.1371/annotation/4c8ccb14-3087-47bf-83a8-b3332d352fe3

**Published:** 2014-01-02

**Authors:** Fang Chen, Juan Shi, You-qing Luo, Shuang-yan Sun, Min Pu

The fifth sentence of the Abstract is incorrect. The sentence should read: "Clustering analysis (using an unweighted pair-group method with arithmetic mean), revealed strong concordance between the strength of genetic relationships among populations and their geographic proximity."

In addition, the second sentence of the first paragraph of the Introduction is incorrect. The sentence should read: Indeed, it is has been listed in the American Quarantine regulations [1]. 

